# Bicyclol: A Useful Agent for Silicosis?

**DOI:** 10.3390/ijms27188212

**Published:** 2026-09-15

**Authors:** Te Zhang, Tong-Tong Liu, Xiao-Jie Wang, Hong-Chao Du, Yan-Xing Han, Yun Zhan, Jian-Dong Jiang

**Affiliations:** State Key Laboratory of Bioactive Substance and Function of Natural Medicines, Institute of Materia Medica, Chinese Academy of Medical Sciences and Peking Union Medical College, Beijing 100050, China; zhangte@imm.ac.cn (T.Z.); liutongtong0315@163.com (T.-T.L.); 19819379095@163.com (X.-J.W.); 18663907895@163.com (H.-C.D.); hanyanxing@imm.ac.cn (Y.-X.H.)

**Keywords:** bicyclol, silicosis, inflammation, fibrosis, ferroptosis, pyroptosis, cellular senescence

## Abstract

Silicosis is one of the most prevalent occupational diseases worldwide, resulting from prolonged exposure to crystalline silica (SiO_2_). This exposure leads to persistent inflammation, lung fibrosis, and ultimately significant loss of lung function and death. Although the precise mechanisms remain unclear, the primary molecular pathogenesis of silicosis—ranging from SiO_2_-induced chronic inflammation to irreversible pulmonary fibrosis—shares considerable overlap with fibrosis mechanisms in various organs, including the secretion of inflammatory cytokines, the activation of fibrosis-related signaling pathways, and ferroptosis. Currently, there is no cure for silicosis; anti-fibrotic treatments are recommended to slow disease progression. Bicyclol, a hepatoprotective agent commonly used to manage elevated aminotransferases due to chronic hepatitis, has garnered attention in recent years for its anti-inflammatory and anti-fibrotic properties in organs such as the liver, heart, and kidney. It significantly reduces extracellular matrix (ECM) synthesis and deposition in these organs, thereby decelerating fibrosis progression. In the context of silicosis, bicyclol has demonstrated promising effects on both chronic inflammation and fibrosis, indicating its potential as a clinical treatment for silicosis. This review summarizes the effects of bicyclol during the chronic inflammation and fibrosis stages of silicosis progression, providing both direct and indirect evidence for its potential therapeutic application in silicosis management. However, it is worth noting that there is limited direct evidence specific to silicosis, and the current prospects mainly stem from indirect evidence of anti-fibrotic effects in other organs. There is still a long way to go before bicyclol truly becomes an approved drug for silicosis.

## 1. Introduction

Silicosis, the most prevalent and detrimental form of pneumoconiosis, remains a significant public health issue in many developing countries, including China, India, and Brazil, primarily due to substandard working conditions. As of 2021, China had reported a total of 915,000 silicosis cases [1]. In 2023, pneumoconiosis continued to be the occupational disease with the highest incidence of new cases, with 8051 new instances reported [2]. Despite rigorous prevention efforts and stringent surveillance implemented in recent years, the number of newly reported cases in China has been decreasing annually. However, given the late-onset nature of silicosis, the country is projected to face an increasing disease burden in the coming years. Alarmingly, this trend is associated with a progressive decline in the average age of onset, with an increasing incidence noted in contemporary occupational sectors such as denim manufacturing, countertop fabrication, and jewelry polishing [3,4]. The latency period of silicosis can range from several months to decades.

Poor-quality chest radiographs present a significant challenge in the early detection of silicosis, particularly in asymptomatic patients, leading to most diagnoses occurring at advanced stages of fibrosis. Pathologically, silicosis progresses through three phases: initial inflammation, the formation of silicotic nodules, and irreversible fibrosis, which ultimately culminates in respiratory failure. This progression is driven by intertwined mechanisms involving chronic inflammation and fibrotic cascades [5]. The Consensus of Chinese Experts on Pneumoconiosis Treatment (2024) recommends that the primary goal of treatment for common clinical symptoms of silicosis be symptomatic relief. For example, cough suppressants, expectorants, and oxygen therapy are utilized to alleviate cough, phlegm, chest tightness, and shortness of breath [6]. However, the medications primarily relieve symptoms and provide only partial improvement in patients’ quality of life. Typically, curative treatment options are limited, with lung transplantation being the only definitive intervention. The development of therapeutic strategies has been impeded by the complex pathogenesis of the disease and an incomplete understanding of its molecular drivers. Nevertheless, oxidative stress, inflammation, and fibrosis are recognized as key factors in the disease’s progression, and targeting each risk factor of silicosis appears to be a promising approach to treatment [5].

**Figure 1:**
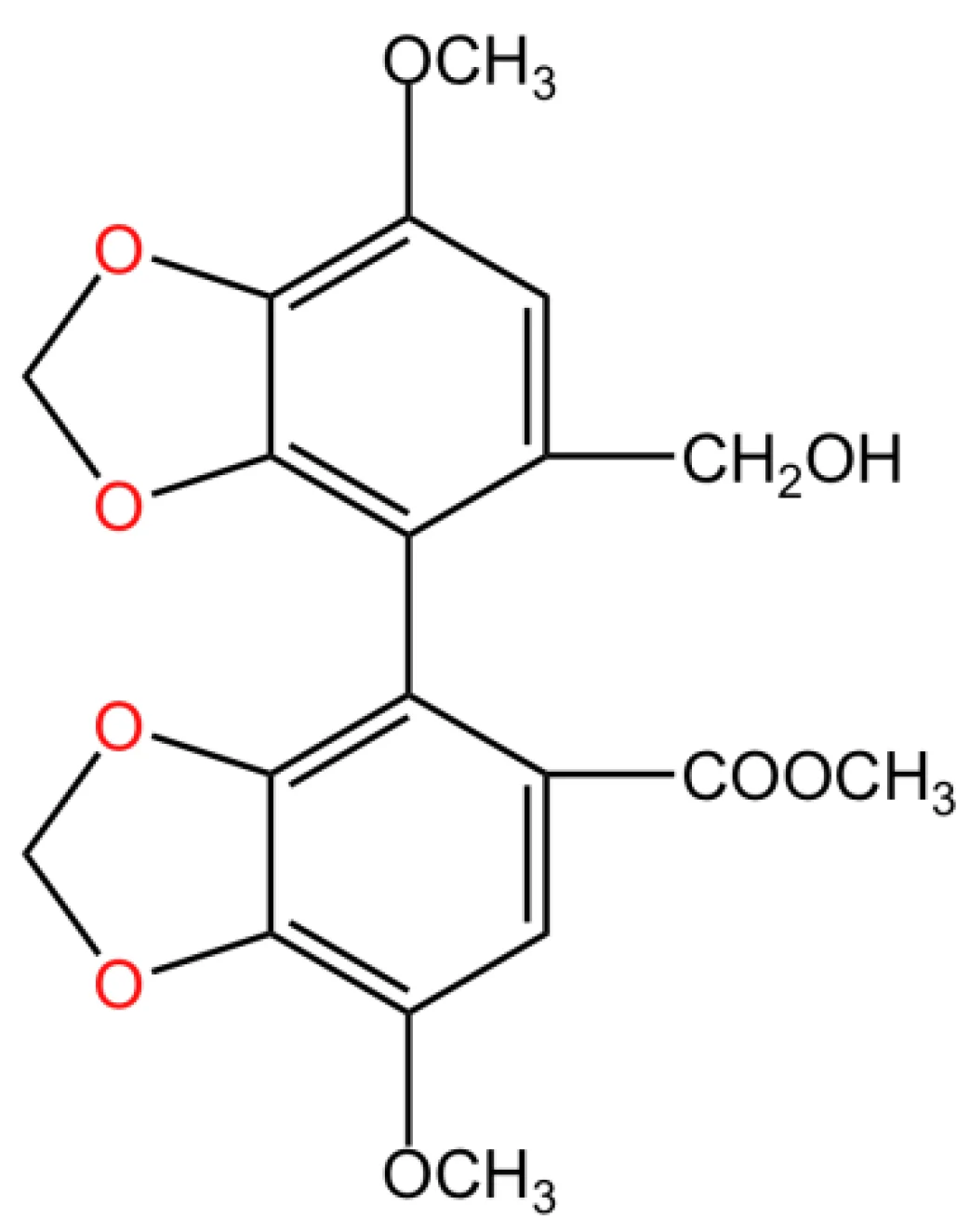
The structure of bicyclol.

Bicyclol (4,4′-dimethoxy-5,6,5′,6′-dimethylene-dioxy-2-hydroxymethyl-2′-carbonyl-biphenyl, Figure 1) is a novel synthetic drug based on the active component, which is extracted from *Fructus schisandrae,* called *schisandrin C*. Due to its effective pharmacological activity and bioavailability in both basic research and clinical applications, as well as its minimal side effects, bicyclol was approved as a hepatoprotective drug by the Chinese Food and Drug Administration (CFDA) in 2004 for the treatment of elevated aminotransferases caused by chronic hepatitis [7]. In the ongoing quest for effective drug therapies related to bicyclol, researchers have expanded their investigative scope beyond liver-related ailments to encompass therapeutic potential for various non-liver diseases and conditions. Bicyclol has been confirmed to exhibit numerous pharmacological activities, including but not limited to anti-inflammatory, anti-oxidant and anti-fibrotic effects. These diverse activities suggest the potential use of bicyclol in treating a wide range of inflammatory and fibrotic conditions, extending beyond those limited to the liver. Indeed, bicyclol has been observed to delay the fibrosis process in multiple organs such as the liver, kidney, and lung in numerous studies, through a multi-target mechanism of action [8,9,10]. Notably, in the context of silicosis, both the anti-inflammatory and anti-fibrotic effects of bicyclol have shown promise in mitigating the progression of pulmonary fibrosis. Therefore, we aim to comprehensively explain the molecular mechanisms by which bicyclol exerts its pharmacological effects in this review and to explore its potential as a drug for silicosis.

## 2. Inflammatory Cascades in Silicosis Pathogenesis

**Figure 2:**
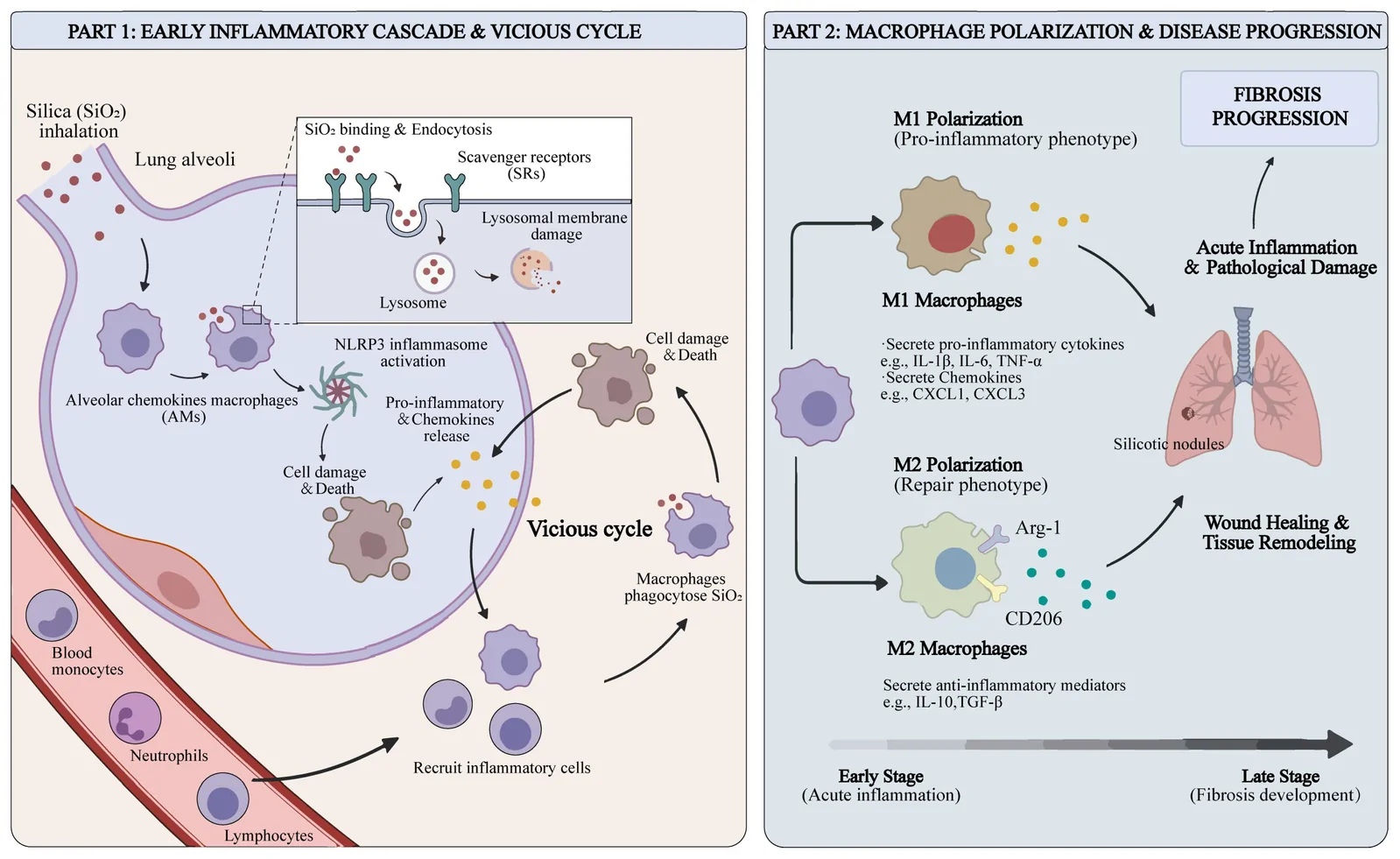
Diagram of the mechanism of silica-induced pulmonary inflammation.

Abundant evidence supports the notion that inflammation plays a critical role in the pathogenesis of silicosis. The inflammation induced by crystalline silica (SiO_2_) exposure is complex and systemic, with macrophage damage and chronic inflammation as the core. The processes and mechanisms underlying this inflammation are illustrated in Figure 2: Upon inhalation of SiO_2_ into the alveoli, alveolar macrophages (AMs) recognize and bind to SiO_2_ particles via the scavenger receptors (SRs) present on their surface. Following this recognition, the particles are internalized into phagolysosomes through endocytosis [11]. During the degradation process, SiO_2_ particles compromise the lysosomal membrane of macrophages, triggering the activation of NOD-like receptor thermal protein domain associated protein 3 (NLRP3) inflammasomes, which can lead to cell damage or death [12]. Subsequently, the damaged macrophages promote the release of pro-inflammatory cytokines such as IL-1β and reactive oxygen species (ROS) into the cytoplasm. Concurrently, the released chemokines (such as IL-8) attract neutrophils, lymphocytes, and additional macrophages, resulting in a gathering of immune cells. The newly recruited macrophages continue to phagocytose particles and subsequently undergo cell death, thereby perpetuating a vicious cycle of inflammation [13].

As the number of recruited macrophages increases, they are activated by mediators at the site of lung injury and polarized into two distinct phenotypes: M1 and M2 macrophages [14]. M1 macrophages are classified as pro-inflammatory and are characterized by acute inflammatory responses mediated by the secretion of pro-inflammatory cytokines (e.g., IL-1β, IL-6, TNF-α) and chemokines (e.g., CXCL1, CXCL3), which collectively amplify silicosis-related inflammation [15,16,17]. In contrast, M2 macrophages exhibit a reparative phenotype, facilitating wound healing and tissue remodeling through anti-inflammatory mediators such as IL-10 and TGF-β. Additionally, M2 macrophages express surface markers including Arg-1 and CD206, which are essential for promoting tissue regeneration and driving fibrotic progression during the later stages of silicosis [18].

**Figure 3:**
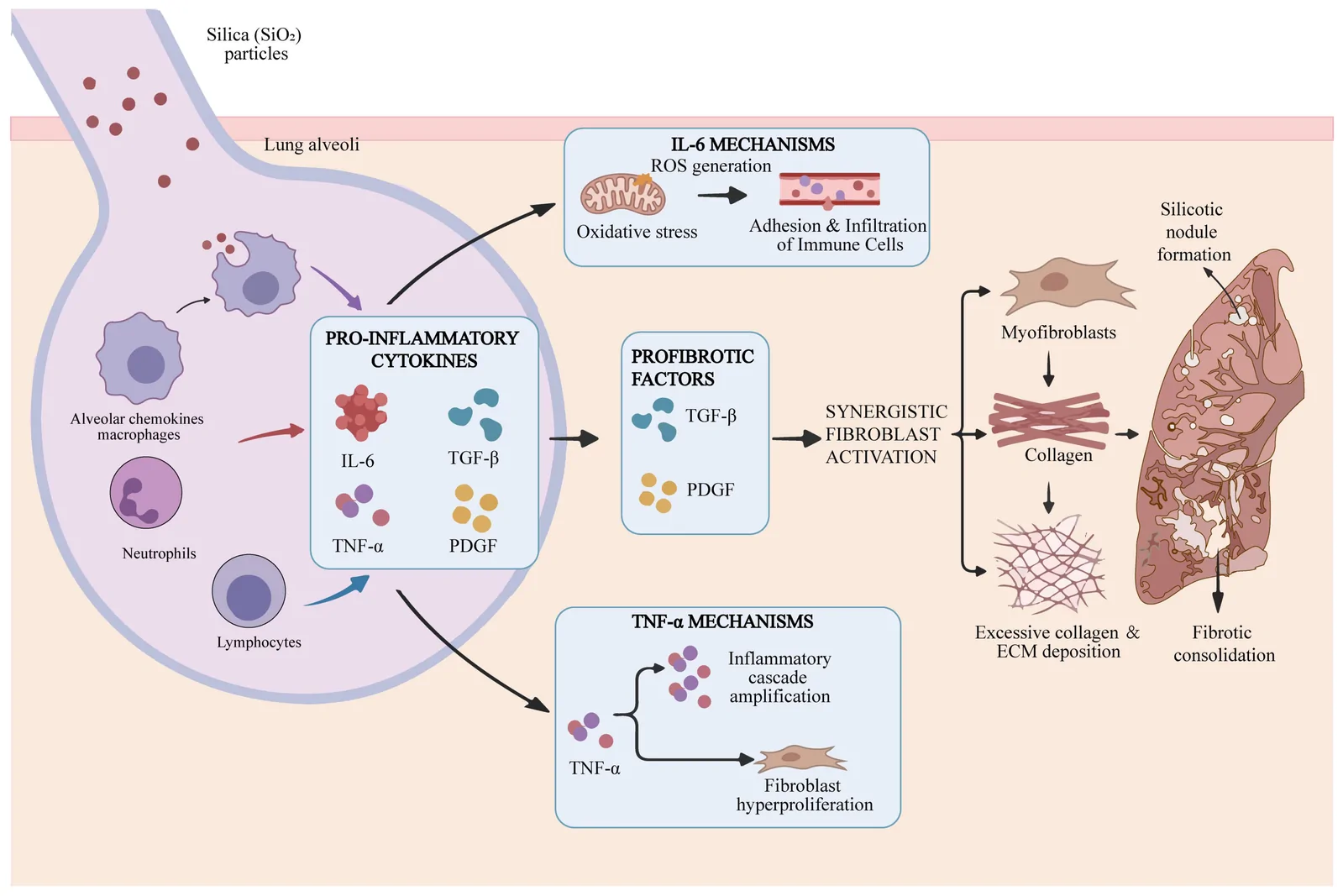
Mechanisms by which other cells and cytokines play a role in the progress of silicosis.

The development of silicosis inflammation involves not only macrophages but also neutrophils, lymphocytes, and other cells. When SiO_2_ is exposed to the lung, it can induce ferroptosis in various lung cells, including alveolar macrophages, type II alveolar epithelial cells, and dendritic cells [14,19,20]. Its core mechanism involves disruption of the Nrf2/SLC7A11/GPX4 axis, leading to lipid peroxidation and iron metabolism disorders. After ferroptosis occurs, it not only promotes the death of various lung cells but also causes damaged cells to release inflammatory factors, further promoting inflammation. Meanwhile, after SiO_2_ is phagocytosed by alveolar macrophages, the particles are recognized by pattern recognition receptors such as Toll-like receptor 4 (TLR4), activating the NLRP3 inflammasome. This process activates caspase-1 to cleave the GSDMD protein, forming cell membrane pores, leading to pyroptosis and the release of a large number of inflammatory factors, which exacerbates lung inflammation [21,22]. Moreover, senescent endothelial cells can secrete Gal3, which also binds to TLR4 on the macrophage membrane, activating the NLRP3 inflammasome [23]. All different signaling pathways contribute to the secretion of pro-inflammatory factors such as IL-6, IL-1β, TNF-α, and platelet-derived growth factor (PDGF) [24]. IL-6 exacerbates dust-induced oxidative stress through ROS generation while enhancing immune cell adhesion and inflammatory infiltration at lesion sites [25]. TNF-α serves as a pivotal mediator in the pathogenesis of silicosis, amplifying inflammatory cascades by stimulating the release of downstream cytokines and directly promoting fibroblast hyperproliferation [26]. What is worse, sustained inflammation perpetuates tissue remodeling; during the inflammatory phase, pro-fibrotic factors such as TGF-β1 and PDGF are concurrently upregulated, synergistically activating fibroblasts and accelerating collagen overproduction and extracellular matrix deposition [27,28]. Collectively, these mediators critically regulate both inflammatory amplification and fibrotic remodeling, contributing to the development of silicotic nodules and subsequent fibrotic consolidation [29] (Figure 3).

## 3. Molecular Mechanisms of Silicotic Fibrosis

Progressive and irreversible silicotic nodules and fibrosis represent the ultimate pathological manifestation of silicosis. Sustained inflammation leads to a massive proliferation and activation of myofibroblasts, which in turn promotes the abnormal deposition of the extracellular matrix (ECM) and results in lung interstitial fibrosis [5]. Ferroptotic cells induced by SiO_2_ release large amounts of damage-associated molecular patterns and pro-fibrotic factors, further activating fibroblasts and promoting their transformation into myofibroblasts. Simultaneously, they disrupt the epithelial barrier, exacerbating ECM deposition and, ultimately, in conjunction with classical pathways, driving irreversible fibrosis progression [14]. Pyroptotic macrophages release exosomes carrying specific miRNAs. These exosomes can be taken up by fibroblasts, promoting their transformation into myofibroblasts and directly driving fibrosis [30]. Pyroptosis also promotes macrophage polarization towards the M2 type and secretion of TGF-β1 [22]. Senescent type II alveolar epithelial cells secrete large amounts of growth differentiation factor 15 (GDF15). As a senescence-associated secretory phenotype factor, GDF15 promotes epithelial–mesenchymal transition (EMT) and activates fibroblasts through the TGF-β receptor [31].

**Figure 4:**
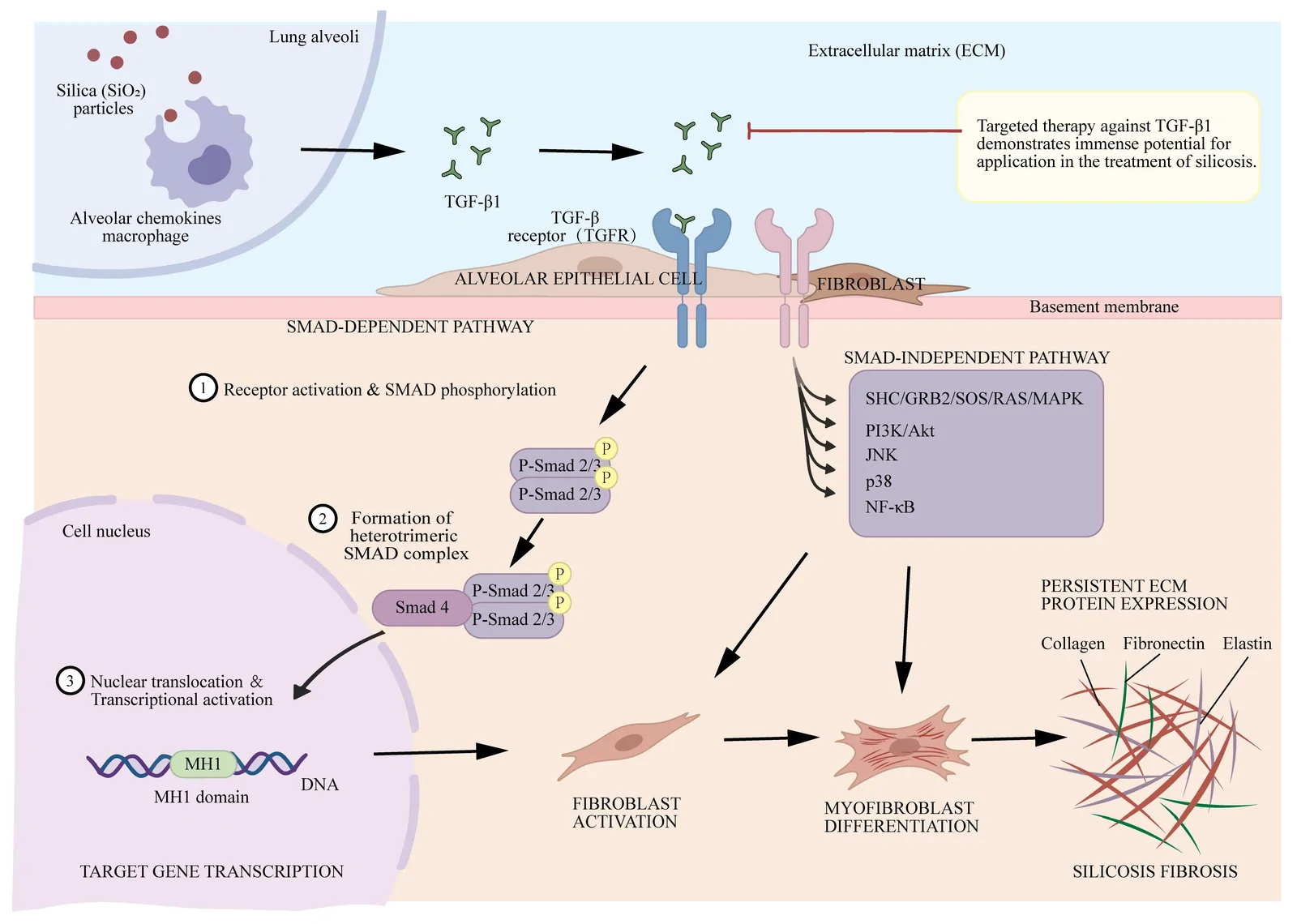
The important role of the TGF-β signaling pathway in silicotic fibrosis.

As mentioned above, the TGF-β family is a crucial group of cytokines implicated in the progression of fibrosis. This family comprises TGF-β1, TGF-β2, and TGF-β3, which are widely expressed in mammals [32]. Among them, TGF-β1 is not only a key signal for initiating fibrosis, but also a core factor that runs through the entire course of the disease and drives multiple pathological processes. Its powerful pro-fibrotic ability and central role in the pathogenesis of silicosis are unmatched by TGF-β2 and TGF-β3. Hence, TGF-β1 is recognized as the most significant factor driving tissue fibrosis [33]. The fibrogenic factor TGF-β1, induced by SiO_2_, is considered a primary contributor to fibrosis in silicosis and plays a vital role in the progression of hepatic, renal, cutaneous, and cardiac fibrosis [34]. TGF-β1 has long been considered a key mediator in fibrosis by activating its downstream SMAD signaling pathway. It has been demonstrated that upon TGF-β1’s action on cells, binding to the TGF-β receptor (TGFR) activates the receptor, leading to the phosphorylation of SMAD2 and SMAD3 at their C-termini. These phosphorylated proteins then form a trimeric SMAD complex with SMAD4, which translocates into the nucleus and binds to DNA through its MH1 domains, triggering the transcription of target genes responsible for the sustained expression of ECM proteins such as collagen, fibronectin, and elastin [35]. Additionally, TGF-β1 activates non-SMAD pathways that regulate fibrosis, including SHC/GRB2/SOS/RAS/MAPK, PI3K/Akt, JNK, p38 and NF-κB [35] (Figure 4). Consequently, the inhibition of TGF-β1 has been shown to mitigate the development of fibrosis in numerous experimental models, and TGF-β1-targeted therapies exhibit considerable potential in the treatment of silicosis [36,37]. Therefore, developing interventions that target the TGF-β1 pathway represents a highly promising anti-fibrotic strategy, although reversing established, advanced consolidation remains a significant clinical challenge.

## 4. Anti-Inflammatory Effects of Bicyclol

**Figure 5:**
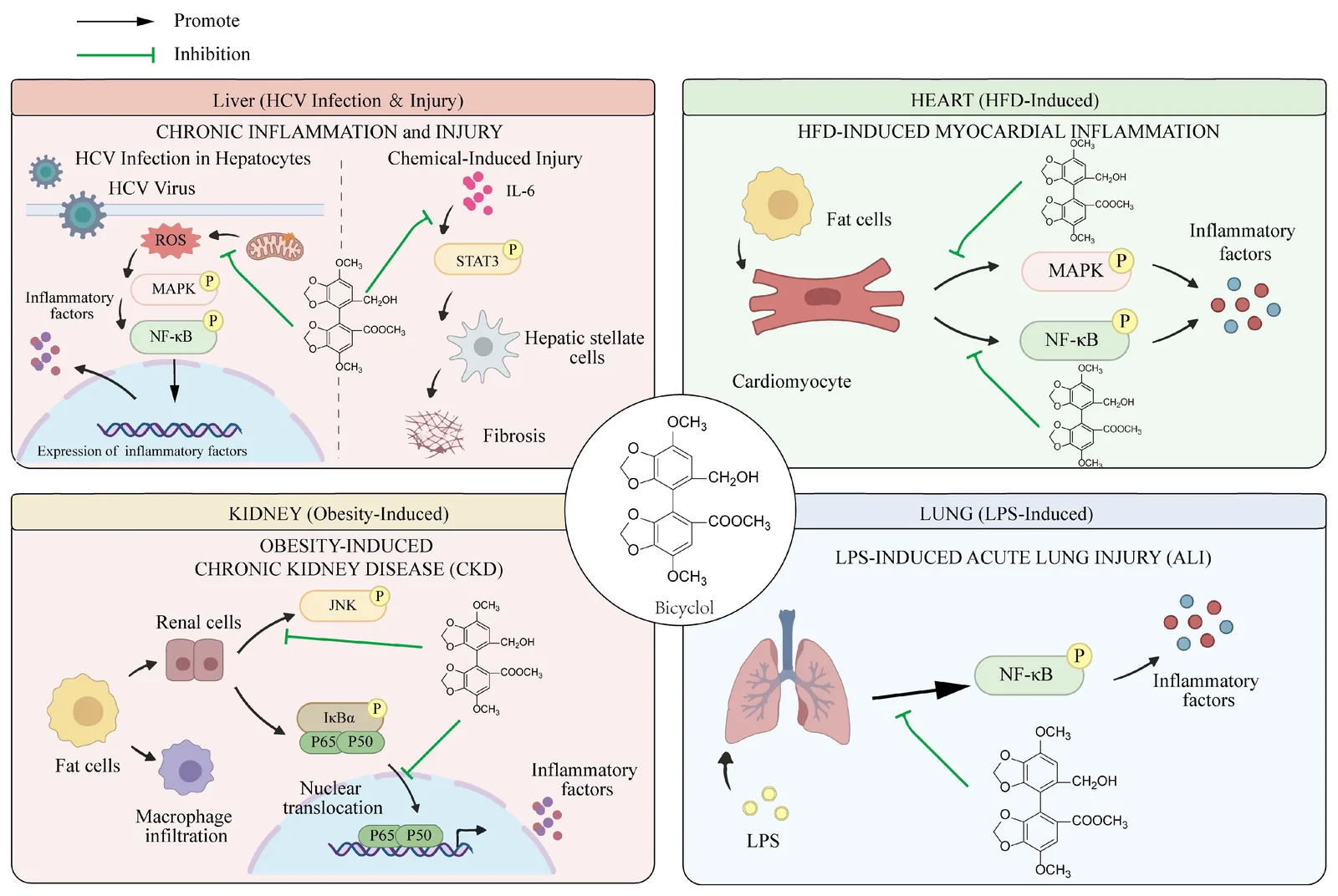
Mechanisms of the therapeutic effects of bicyclol in the treatment of inflammation in the heart, liver, kidney, and lung.

Numerous studies have demonstrated that bicyclol exerts significant anti-inflammatory effects across various organs (Figure 5). In the liver, bicyclol ameliorates virus-induced chronic inflammation by inhibiting the activation of the ROS-MAPK-NF-κB signaling pathway, thereby reducing NF-κB activity and the mRNA levels of inflammatory cytokines, such as TNF-α, IL-6, and MIP-1β, within hepatocytes infected with the hepatitis C virus [38]. In the heart, bicyclol significantly attenuates obesity-induced myocardial inflammatory injury by inhibiting the MAPK and NF-κB pathways, thereby suppressing the high-fat diet-induced elevation of inflammatory cytokines such as IL-1β, IL-6, and TNF-α [39]. Similarly, in obesity-induced chronic kidney disease, bicyclol reduces macrophage infiltration and the expression of pro-inflammatory factors (IL-6, TNF-α, MMP-2, MMP-9, VCAM-1) by inhibiting the JNK and NF-κB pathways [9]. In lipopolysaccharide (LPS)-induced acute lung injury, bicyclol prevents the release of inflammatory factors such as TNF-α, IL-6, and IL-1β by inhibiting the activation of NF-κB mediated by MyD88, which is accompanied by an improved survival rate of mice [40]. Therefore, bicyclol mainly exerts anti-inflammatory effects via inhibiting multiple inflammatory signaling pathways, resulting in the down-regulation of pro-inflammatory cytokines such as IL-6 and TNF-α.

Previous studies have indicated that various cytokines, including TGF-β1, IL-6, TNF-α, and IL-1β, are key factors in the development of pulmonary fibrosis in relation to silicosis [41]. Among them, inflammatory cytokines IL-6, TNF-α and IL-1β, serve as indicators of inflammation [42,43,44]. Most therapeutic agents that are effective in pulmonary fibrosis models characteristically attenuate the secretion of these pro-inflammatory cytokines [5]. Notably, bicyclol significantly suppresses the secretion of inflammatory cytokines IL-1β, IL-6, and TNF-α in both bronchoalveolar lavage fluid (BALF) and lung tissue of silicosis rats [37]. This provides the most direct and compelling evidence for the anti-inflammatory effect of bicyclol in the treatment of silicosis.

Unlike the inflammatory signals inhibited by bicyclol in other organs, in vitro experiments demonstrate that bicyclol reduces the mRNA and protein levels of inflammatory cytokines in a dose-dependent manner in SiO_2_-stimulated RAW264.7 cells via inhibiting the JAK2/STAT3/SOCS3 signaling pathway, which was confirmed in the in vivo silicotic rat model [37]. Of course, this should not be the only signaling pathway that bicyclol inhibits in silicosis. After all, following exposure to silica, NF-κB translocates into the nucleus and initiates the transcription of TNF-α, IL-1β, and IL-6 [45,46]. Meanwhile, the MAPK/ERK pathway regulates the expression of genes associated with inflammation and fibrosis, including PDGF and ICAM-1 [47]. Further data are needed to confirm that these signaling pathways are regulated by bicyclol in silicosis, through which bicyclol exerts its anti-inflammatory effects in other organs. However, it is undeniable that bicyclol can reduce the expression of the main inflammatory factors in silicosis.

**Figure 6:**
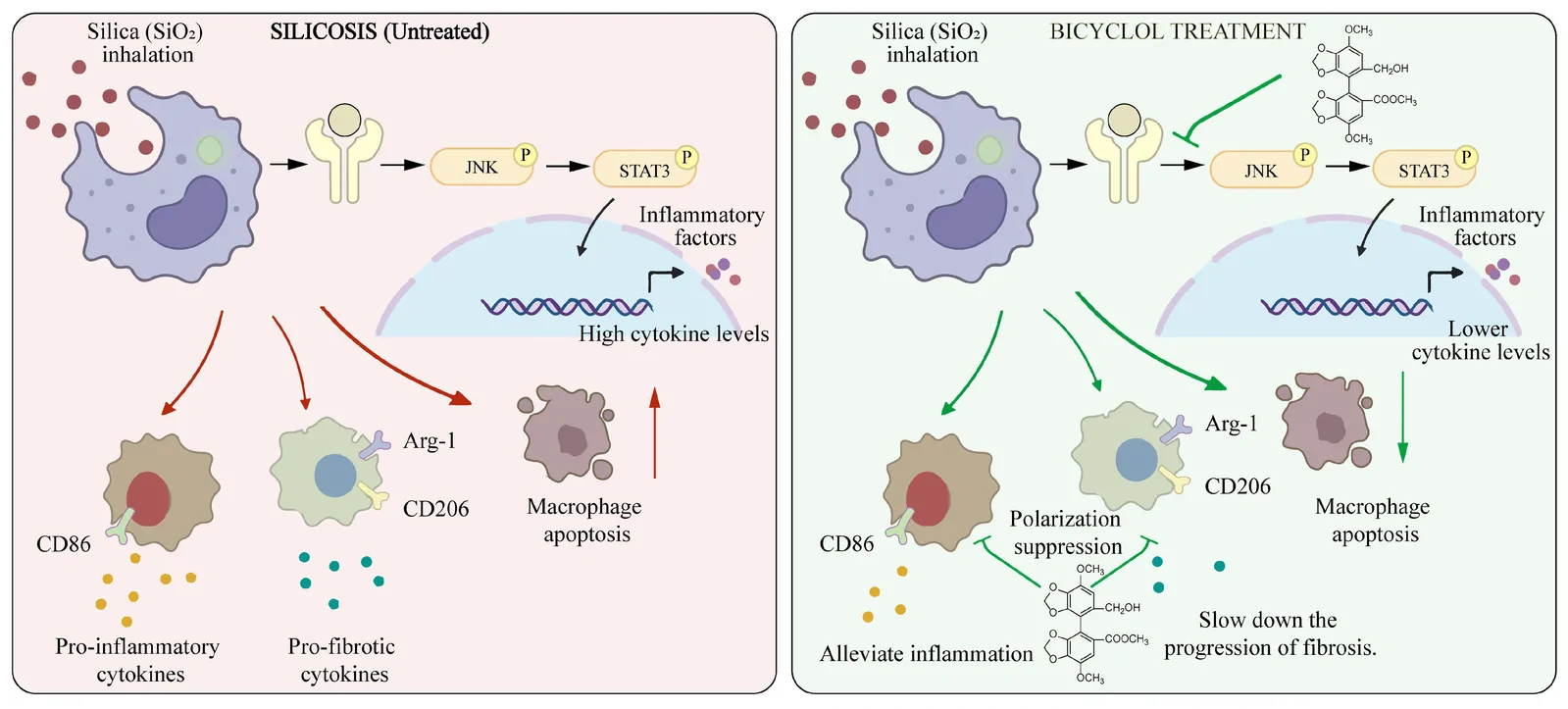
The mechanism of the therapeutic effect of bicyclol on silicosis-induced inflammation.

As detailed in the pathogenesis of silicosis, SiO_2_ exposure triggers NLRP3 inflammasome activation, pyroptosis, and ferroptosis, alongside macrophage M1/M2 polarization, all of which contribute to a vicious cycle of inflammation. It is reported that bicyclol reduced the accumulation of inflammatory cells in the lungs and protected cells against SiO_2_-induced apoptosis, thereby interrupting the macrophage cascade reaction [37,48]. Across both in vitro and in vivo silicosis models, bicyclol treatment significantly downregulated the expression of M1 macrophage markers (iNOS and CD86) as well as M2 markers (Arg-1 and CD163) [37,48]. These findings indicate that bicyclol effectively suppresses both pro-inflammatory M1 and pro-fibrotic M2 macrophage polarization, suggesting that bicyclol may alleviate lung inflammation and delay the progression of silicosis through these mechanisms (Figure 6).

It should be noted that existing studies have identified the regulation of bicyclol in the secretion of pro-inflammatory cytokines. However, its role in novel pathological processes such as NLRP3 inflammasome activation, pyroptosis, and ferroptosis remains inadequately explored, with a lack of systematic functional validation and mechanistic insights. Future targeted research aimed at clarifying the specific molecular targets and regulatory networks of bicyclol will provide stronger theoretical support for its clinical application in anti-inflammatory therapy for silicosis.

## 5. Anti-Fibrotic Effect of Bicyclol

Research indicates that drugs reducing inflammation may be effective in preventing silicosis. Notably, certain medications targeting inflammatory responses, such as anakinra (an IL-1 receptor antagonist), infliximab (a TNF-α inhibitor), and glucocorticoids, have demonstrated efficacy [5]. Given the alleviation of inflammatory symptoms in both silicosis and other diseases by bicyclol, it is proposed that bicyclol may possess the potential to decelerate the progression of silicosis through its anti-inflammatory properties. However, relying solely on anti-inflammatory therapy is insufficient to impede the progression of silicosis and may even result in adverse clinical outcomes for patients with fibrotic lung disorders [37], indicating that bicyclol possesses other properties relevant to the treatment of silicosis besides anti-inflammation.

The Consensus of Chinese Experts on Pneumoconiosis Treatment (2024) emphasizes the importance of anti-fibrosis therapy in the treatment of silicosis, identifying it as one of the recommended therapeutic approaches [49]. As a multi-target drug, bicyclol has been demonstrated to have significant inhibitory effects on liver fibrosis, myocardial fibrosis, and renal interstitial fibrosis. Bicyclol inhibited bovine serum albumin (BSA)-induced rat liver fibrosis by suppressing the TGF-β1/SMAD2/3 signaling pathway, thereby maintaining the balance between ECM deposition and degradation [50]. A randomized controlled trial involving 80 patients with chronic hepatitis B showed that bicyclol significantly reduced the synthesis and deposition of fibrotic markers such as Col1a1, Col3a1, hyaluronic acid, and laminin [51]. In a model of diabetic cardiomyopathy, bicyclol not only mitigated cardiac injury and myocardial hypertrophy but also significantly downregulated the mRNA and protein levels of fibrosis-related genes TGF-β1 and Col1a1, thereby inhibiting connective tissue hyperplasia [52]. Similarly, in a mouse model of renal fibrosis induced by a high-fat diet, treatment with bicyclol reduced collagen deposition and reversed the upregulation of Col1a1 and TGF-β1 expression caused by the high-fat diet [9]. Collectively, these findings provide a theoretical basis for considering bicyclol as a potential therapeutic agent for silicosis.

Although the lung differs from the liver, heart, and kidney in terms of anatomical structure and microenvironment, the core pathological mechanisms of pulmonary fibrosis—namely, damage to alveolar epithelial cells, abnormal activation of fibroblasts, and excessive collagen deposition—are also reliant on the aberrant activation of similar signaling pathways [5,8]. Consequently, the anti-fibrotic effect of bicyclol may exhibit cross-organ universality. Studies have demonstrated that in the bleomycin-induced idiopathic pulmonary fibrosis (IPF) rat model, the extent of fibrosis and collagen deposition in the bicyclol treatment group was significantly reduced, with the IPF score being markedly lower than that of the vehicle group (*p* < 0.05). This finding substantiates the inhibitory effect of bicyclol on pulmonary fibrosis in rats [8], and suggests its potential in treating silicosis-related fibrosis. However, IPF and silicosis fundamentally differ in their etiology and early immuno-inflammatory cascades. IPF primarily stems from recurrent alveolar epithelial injury and aberrant repair, whereas silicosis is driven by continuous macrophage stimulation and chronic inflammation induced by silica dust. Consequently, extrapolating therapeutic efficacy to silicosis solely from other organ systems or IPF models warrants caution, underscoring the urgent need for direct evidence from silicosis-specific pathological models. Hence, it is further confirmed that bicyclol administration reduced collagen synthesis and deposition in lung tissues and significantly improved lung function parameters, including static compliance, expiratory volume, and lung stiffness, in a silica-induced rat model of silicosis. The expression of ECM components, such as Col1a1, Col3a1, and fibronectin, was significantly diminished by bicyclol both in vitro and in vivo [37]. These findings provide direct evidence to support the effectiveness of bicyclol in silicosis.

**Figure 7:**
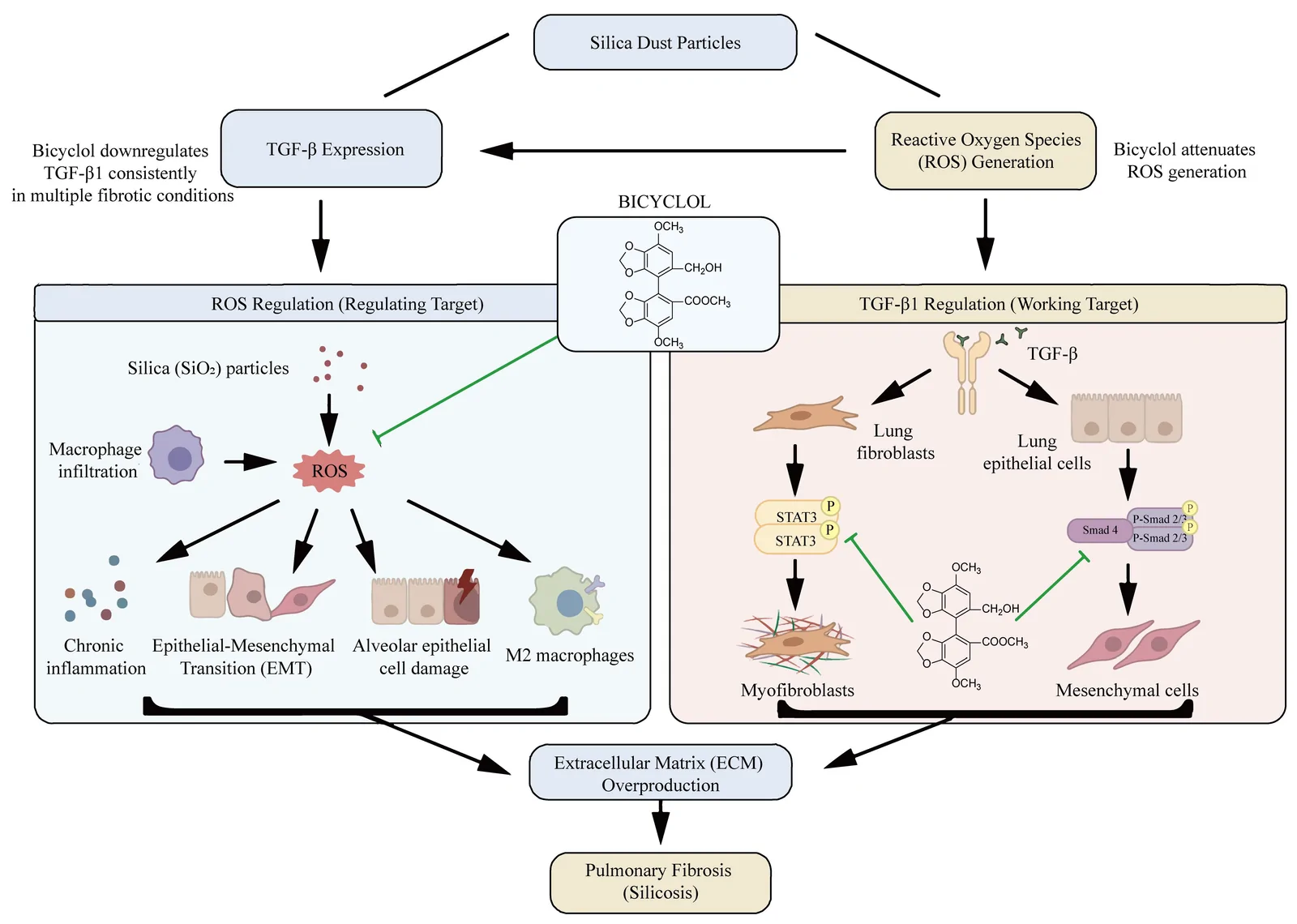
The mechanism of the therapeutic effect of bicyclol on silicotic pulmonary fibrosis.

It is widely considered that TGF-β1 plays a critical role in the process of fibrosis. Bicyclol has been identified to down-regulate the expression of TGF-β1 in the process of multi-organ fibrosis [9,50,52]. In a bleomycin-induced pulmonary fibrosis model, bicyclol down-regulated the expression of TGF-β1, leading to the diminishment of fibrosis [53]. Not surprisingly, bicyclol was observed to significantly inhibit TGF-β1 and its transcription levels in silicosis. At the level of downstream target cells, bicyclol suppresses EMT of pulmonary epithelial cells by blocking the canonical TGF-β1 pathway, while simultaneously inhibiting the fibroblast-myofibroblast transformation (FMT) by blocking the non-canonical pathway, ultimately mitigating the abnormal accumulation of ECM [37]. This further confirmed the anti-fibrotic effect of bicyclol in silicosis. Although it is undeniable that bicyclol reduces the expression of TGF-β1 during the process of silicosis fibrosis, it is still unclear whether this reduction is caused by the direct interaction between bicyclol and TGF-β1, or whether it is indirectly triggered by other factors. Notably, the burst of ROS induced by silica dust is one of the main triggers driving the progression of silicosis and promoting the secretion of TGF-β1. ROS derived from alveolar cells and macrophages not only cause direct tissue damage, but also induce macrophage polarization toward the M2 phenotype and promote the secretion and activation of TGF-β1 [54,55,56,57,58]. Across multiple organ protection models—including the brain, liver, and heart—bicyclol has demonstrated the capacity to scavenge ROS by activating anti-oxidant pathways [59,60,61]. Therefore, attenuating ROS-mediated oxidative stress to interrupt the ROS-macrophage activation-TGF-β1 secretion-FMT/EMT cascade likely represents one of the core mechanisms by which bicyclol suppresses silicosis fibrosis progression (Figure 7).

Overall, based on the widespread downregulation of TGF-β1 and ECM expression in multi-organ fibrosis and recent prospective studies specific to silicosis, bicyclol shows preclinical promise as a candidate therapeutic agent for anti-fibrotic treatment in silicosis. It needs to be emphasized that the ability to reduce the expression of TGF-β1 and ECM only indicates the potential of bicyclol for treating silicosis. While the anti-fibrotic evidence of bicyclol in liver, kidney, and cardiac fibrosis models provide directional clues for silicosis research, its extrapolation value has clear limitations. First, the initiating factors, core effector cells, and microenvironmental regulatory networks of fibrosis differ across organs. For example, silicosis is characterized by continuous silica stimulation and alveolar macrophage activation, while liver fibrosis is centered on hepatic stellate cell activation. Heterogeneous expression of shared pathways may affect drug efficacy. Second, direct data on the distribution concentration, target binding efficiency, and pharmacokinetic characteristics of bicyclol in lung tissue are lacking, and its accessibility to target cells in the lung cannot be simply replaced by liver tissue data. Finally, existing animal models cannot fully reproduce the chronic, progressive pathological process of human silicosis, and differences in immune response and fibrosis progression between animals and humans further weaken the translatability of indirect evidence into certainty. Therefore, the aforementioned cross-organ evidence should be characterized as exploratory clues rather than definitive evidence of clinical efficacy. Whether bicyclol can truly be used for clinical treatment of silicosis still requires more supporting data.

## 6. Potential Regulatory Role of Bicyclol in Silicosis

As mentioned above, silica can cause the death of various lung cells, such as alveolar macrophages and epithelial cells, through the Nrf2/SLC7A11/GPX4 signaling axis, thereby releasing inflammatory signals and promoting key pathways in pulmonary fibrosis. However, the hypothesis that bicyclol directly modulates these specific cell death pathways in silicosis remains a scientific conjecture, because current support comes almost exclusively from indirect evidence obtained in other disease models. For example, bicyclol has been shown to protect against ferroptosis in CCl4-induced acute liver injury by clearing ROS, restoring mitochondrial function, and upregulating GPX4 expression levels [62]. Based on this, bicyclol may exert a potential anti-ferroptosis effect in silicosis. Furthermore, in obesity-related nephropathy, bicyclol can block pyroptosis signaling by inhibiting the NF-κB pathway, thereby reducing NLRP3 inflammasome activation. It also inhibits the JNK pathway, alleviating the inflammatory environment and synergistically reducing fibrosis levels [9]. Unfortunately, there are currently no reports on bicyclol directly inhibiting cellular senescence. However, the anti-oxidant and anti-inflammatory properties of bicyclol play a role in protecting cells and reducing damage, which may indirectly delay or improve cellular senescence phenotypes related to oxidative stress and inflammation.

However, this inference has several limitations, such as the lack of direct evidence that bicyclol directly targets the core regulatory axis of ferroptosis and pyroptosis in silicosis models. Furthermore, while the multi-target nature of bicyclol offers synergistic therapeutic potential, it also makes it difficult to clearly distinguish whether the inhibition of ferroptosis and pyroptosis is a direct pharmacological effect or an effect secondary to the improvement of the overall inflammatory microenvironment. In summary, although the current evidence is indirect, the scientific hypothesis that bicyclol intervenes in ferroptosis in silicosis by regulating redox balance has a solid theoretical foundation. With further in-depth analysis of the above mechanism regulatory network, bicyclol or its derivatives are expected to become highly promising candidate drugs for the comprehensive treatment of silicosis.

## 7. Safety of Bicyclol

According to the Consensus of Chinese Experts on Pneumoconiosis Treatment (2024), two anti-fibrotic drugs, tetrandrine and nintedanib, have been recommended for silicosis treatment in an oral form. Experimental studies demonstrate that tetrandrine inhibits the progression of silicosis through multiple mechanisms, including suppressing fibroblast metabolism and inhibiting collagen synthesis. A clinical trial involving 89 patients with silicosis revealed that three months of tetrandrine treatment reduced the progression rate from 92% to 5.3%. Compared with the control group, nintedanib delayed the annual rate of forced vital capacity decline by 107 mL in the experimental group and significantly reduced the risk of acute exacerbation or death in progressive fibrosing interstitial lung disease patients by 33%. In addition, in a subgroup cohort of 39 patients with occupational exposure-related interstitial lung disease, the difference in the annual rate of forced vital capacity decline between the two groups was 252.8 mL [49].

Given the similarities between IPF and silicosis, pirfenidone, an anti-fibrotic drug for IPF, is being considered for the treatment of silicosis [63]. It has been demonstrated that pirfenidone significantly improved pulmonary dysfunction, lung inflammation, and pulmonary fibrosis in a mouse silicosis model [64]. It is worth mentioning that pirfenidone is currently undergoing a Phase III clinical trial for the treatment of silicosis (NCT05288179). Similarly, bicyclol effectively reduced the number of inflammatory cells and the release of inflammatory factors in a rat silicosis model, while also decreasing extracellular matrix deposition [37], demonstrating a role in slowing the progression of silicosis and suggesting potential use in clinical silicosis therapy.

However, the toxicity and side effects associated with these drugs significantly restrict their usage. The most common adverse effect of tetrandrine is hepatotoxicity, characterized by elevated levels of aspartate aminotransferase (AST) and alanine aminotransferase (ALT), which can lead to liver damage [65]. Nintedanib is associated with severe adverse reactions, including diarrhea, nausea, vomiting, abdominal pain, loss of appetite, and elevated transaminases, with a notable risk of hepatotoxicity [66]. Furthermore, pirfenidone has demonstrated efficacy in treating silicosis [64], yet it is linked to adverse effects such as IPF progression, upper respiratory tract infection, bronchitis, nausea, diarrhea, and hepatotoxicity [67,68]. Bicyclol may occasionally cause side effects such as rash, dizziness, nausea, headache, and pruritus. Data from the National Drug Adverse Drug Reaction Monitoring System, covering the period from 2013 to 2017, showed that the number of people using bicyclol was 500,000 to 1.2 million per year. The incidence of adverse reactions associated with bicyclol tablets remains stable at approximately one in ten thousand. Additionally, among the 358 reported adverse drug reactions, a significant majority (85.7%) of symptoms resolved after discontinuation of the drug [69]. Notably, bicyclol was initially approved as a hepatoprotective drug by the CFDA due to its efficacy in reducing ALT and AST levels. This characteristic enables bicyclol to offer supplementary protection in the treatment of liver injury [70,71]. It should be pointed out that the above adverse reaction data are from different clinical trials and real-world studies, with varying patient populations, medication regimens, and monitoring methods. Specific comparisons of adverse event data between real-world studies and clinical trials of these drugs should be made with caution (Table 1).

Bicyclol is recommended with an oral dosage of 25 mg three times daily in China. In healthy subjects, the peak plasma concentration after oral administration is approximately 50 ng/mL, with a time to peak of about 1.8 h and an elimination half-life of approximately 6.3 h. The drug exhibits linear pharmacokinetic characteristics within the dosage range of 25–100 mg, and there is no accumulation after multiple administrations [72]. However, its low oral bioavailability is primarily attributed to intestinal P-glycoprotein efflux and CYP3A-mediated first-pass metabolism, which may limit the effective exposure of the drug in the lungs [79]. Currently, animal experiments on silicosis often employ gavage administration, but this dosage is significantly higher than the conventional human dosage, and there is a lack of data supporting a direct correlation between local drug concentration in the lungs and drug efficacy [48]. The high-dose gavage administration of bicyclol in animal studies has unclear equivalence to standard clinical doses, and its low oral bioavailability may limit effective lung exposure, constituting a limitation in the interpretation of current evidence. It is suggested that if bicyclol is repurposed for the treatment of silicosis, it is crucial to address whether the current oral regimen is optimal and whether strategies such as local pulmonary drug delivery can be explored. Overall, bicyclol warrants special attention as a potential drug candidate for silicosis, due to its established safety profile from long-term clinical use, proven multi-organ anti-fibrotic activity, and emerging evidence of efficacy in preclinical models of silicosis.

## 8. Conclusions

Synthesizing findings from existing literature, researchers have found that bicyclol has been demonstrated to have clear anti-inflammatory and anti-fibrotic effects in both in vitro models and in vivo animal experiments. Therefore, in terms of mechanism, bicyclol has a certain pharmacological basis for alleviating the progression of silicosis in rats and holds the possibility of alleviating silicosis in humans.

Beyond its direct anti-inflammatory and anti-fibrotic actions, emerging evidence suggests that bicyclol may also modulate the gut microbiota and intestinal barrier function [80]. Meanwhile, recent studies have demonstrated that regulating the gut microbiota via fecal microbiota transplantation or *Bifidobacterium* supplementation can attenuate silicosis fibrosis through the gut-lung axis [81]. Given that both pathways converge on the TGF-β1/Smad signaling cascade, bicyclol might indirectly exert its anti-silicotic effects by remodeling the gut microbial community and increasing short-chain fatty acid production, thereby suppressing TGF-β1 signaling. However, this hypothesis requires experimental validation using strategies such as fecal microbiota transplantation and antibiotic depletion.

As a drug that has been on the market for many years, bicyclol can save a lot of time and costs from the perspective of developing new drugs for the treatment of silicosis, which is another advantage of bicyclol. However, addressing the core question of whether bicyclol can serve as a viable therapeutic agent for silicosis requires bridging the gap between preclinical data and clinical translation. For example, in animal experiments, there is an urgent need for more animal model validation for silicosis, such as determining the time window for administration of bicyclol and drug concentration in lung tissue. Meanwhile, its main bottleneck lies in the lack of large-scale, multi-center clinical trial data and conclusive evidence of its anti-fibrotic mechanism in the human body.

Given the lack of direct clinical evidence for bicyclol in silicosis patients, future early clinical trials should incorporate pharmacodynamic biomarkers into their study designs. Classic biomarkers such as KL-6, SP-D, CC16, and inflammatory factors such as TNF-α and TGF-β1 have been widely used to assess epithelial damage and fibrotic activity in silicosis [82,83,84]. In recent years, new biomarkers such as exosomal miRNAs (e.g., miR-23a-3p), highly specific proteins such as OC-STAMP, and dual-protein markers have emerged. These biomarkers can reflect early abnormalities in epithelial apoptosis and fibrosis processes, endoplasmic reticulum stress levels, and circulating chemokine profiles, providing more precise tools for early diagnosis and mechanistic research [14,85,86]. Dynamic changes in these biomarkers can provide early signals of the biological effects of bicyclol and a basis for dose selection and endpoint determination in subsequent confirmatory trials. Simultaneously, proof-of-concept can be conducted by combining short-term endpoints such as forced vital capacity, diffusing capacity of the lung for carbon monoxide, and six-minute walk test, while later clinical trials use annual decline rate of lung function, radiological progression, and composite clinical outcomes as endpoints [49,87].

Current studies predominantly utilize oral administration, and due to the hepatic first-pass effect, localized drug concentrations in the lungs may be constrained. It is necessary to clarify the possibility of improving the formulation of bicyclol, as well as the targeted distribution and bioavailability of different formulations of bicyclol in lung tissue. Developing an inhalable formulation of bicyclol could deliver the drug directly to affected pulmonary regions, thereby enhancing localized anti-fibrotic efficacy while further mitigating systemic adverse effects. Notably, inhalable nintedanib formulations have already demonstrated superior pulmonary delivery efficiency and higher tissue concentration in mouse models, significantly enhancing bioavailability [88], which also provides a new approach for the application of bicyclol in the treatment of silicosis.

Additionally, this review has several limitations. The primary conclusions rely heavily on data from rodent models and in vitro cellular assays, which fail to fully recapitulate the chronic pathological progression of human silicosis that typically spans years or decades. Furthermore, an accurate translation mechanism remains lacking to reconcile preclinical dosage regimens with human safe and effective long-term dosing. Future research should prioritize evaluating the pharmacokinetics and pulmonary toxicology of inhalable bicyclol, alongside exploring combination therapy strategies that pair bicyclol with existing anti-fibrotic agents or whole-lung lavage procedures.

In summary, bicyclol has demonstrated significant therapeutic effects on both in vitro and in vivo silicosis models, particularly in terms of its anti-inflammatory and anti-fibrotic properties. Additionally, bicyclol exhibits a low incidence of adverse reactions, suggesting its potential for long-term medication. Although existing data do not support the use of bicyclol as a substitute for mainstream anti-fibrotic drugs, its preliminary safety data and observed multi-organ anti-fibrotic activity indicate that it possesses the potential to become a therapeutic candidate for silicosis. Given that current evidence is derived from a single team and lacks independent validation, future research urgently needs multi-center, large-sample animal experiments, and should prioritize well-designed randomized controlled clinical trials to clarify the true efficacy and safety of bicyclol in the treatment of silicosis.

## Figures and Tables

**Table 1:** Comparison of safety between bicyclol and existing anti-fibrotic drugs.

Drugs	Oral Dosage and Frequency of Oral Administration	Efficacy	Common Adverse	Reactions Hepatotoxicity Risk
Bicyclol	25 mg three times daily [72]	No clinically relevant data available	Extremely low (0.01%) [69]	Protects the liver (reducing ALT and AST levels) [73]
		Delay inflammation and fibrosis progression in the silicosis rat model [37]	Reversible upon discontinuation [69]	
Tetrandrine	60–100 mg three times daily [6]	Delay the progression of silicosis [49]	Hepatotoxicity [65]	Risk of hepatotoxicity limits its clinical application [74]
				Liver toxicity has been observed multiple times in animal experiments [75]
Nintedanib	150 mg two times daily [6]	Delays the annual decline rate of forced vital capacity [49]	Diarrhea, nausea, vomiting [66]	Incidence of elevated liver enzymes up to 14% [76]
Pirfenidone	801 mg three times daily, following a 14-day dose titration [77]	No clinically relevant data available	Upper respiratory tract infection, bronchitis, nausea [67,68]	Non-serious and serious cases of liver injury [78]
		Delay inflammation and fibrosis progression in the silicosis mouse model [64]		Severe liver injury with fatal outcome [78]

## Data Availability

No new data were created or analyzed in this study. Data sharing is not applicable to this article.

## Abbreviations

The following abbreviations are used in this manuscript:

ALT	Alanine aminotransferase
AMs	Alveolar macrophages
AST	Aspartate aminotransferase
BALF	Bronchoalveolar lavage fluid
CFDA	Chinese Food and Drug Administration
ECM	Extracellular matrix
EMT	Epithelial–mesenchymal transition
FMT	Fibroblast-myofibroblast transformation
HSCs	Hepatic stellate cells
iNOS	inducible Nitric Oxide Synthase
IPF	Idiopathic pulmonary fibrosis
LPS	Lipopolysaccharide
NLRP3	NOD-like receptor thermal protein domain associated protein 3
ROS	Reactive oxygen species
SiO_2_	Crystalline silica
SRs	Scavenger receptors
TGFR	TGF-β receptor
TLR4	Toll-like receptor 4
